# Lassa virus circumvents macrophage and dendritic cell antiviral defences in its natural reservoir, the Natal multimammate mouse (*Mastomys natalensis*)

**DOI:** 10.1038/s44298-026-00177-6

**Published:** 2026-02-09

**Authors:** Nicolas Corrales, David M. Wozniak, Ivet A. Yordanova, Ellen L. Suder, Ariadna E. Morales, Angelika Lander, Katharina Hansen-Kant, Michael Hiller, Joseph B. Prescott

**Affiliations:** 1 Centre for Biological Threats and Special Pathogens, Robert Koch Institute, Berlin, Germany; 2 Department of Virology, Immunology and Microbiology, Chobanian & Avedisian School of Medicine, Boston University, Boston, MA USA; 3 National Emerging Infectious Diseases Laboratories (NEIDL), Boston University, Boston, MA USA; 4 Senckenberg Research Institute, Frankfurt (Main), Germany; 5 Institute of Cell Biology and Neuroscience, Faculty of Biosciences, Goethe University Frankfurt, Frankfurt (Main), Germany; 6 ISEM, University of Montpellier, CNRS, IRD, Montpellier, France; 7 Present Address: Bernhard Nocht Institute for Tropical Medicine, Hamburg, Germany

**Keywords:** Immunology, Microbiology

## Abstract

Lassa virus (LASV) is a zoonotic arenavirus that causes severe hemorrhagic fever in humans but persists asymptomatically in its natural reservoir, the Natal multimammate mouse (*Mastomys natalensis*, NMM). The mechanisms underlying this disease tolerance remain poorly understood. Here, we establish and characterize macrophages (bmMΦs) and dendritic cells (bmDCs) derived from NMM bone marrow, and assess their response to LASV infection. Both cell types are permissive to LASV and remain viable throughout infection. However, LASV fails to induce transcriptional activation of antiviral or maturation-associated genes in either cell type, in stark contrast to the robust and stimulus-specific responses elicited by LPS, Poly I:C, and Sendai virus. A modest increase in CD80 surface expression was observed in LASV-infected DCs, uncoupled from transcriptional induction, suggesting post-transcriptional modulation. These data reveal a striking circumvention of innate immune activation in NMM myeloid cells despite productive LASV infection, supporting a model in which early immune evasion facilitates reservoir tolerance. This work provides mechanistic insight into LASV–host co-adaptation and establishes a functional in vitro system for dissecting immune responses in the natural reservoir.

## Introduction

Lassa virus (LASV), an arenavirus endemic to West Africa, is the causative agent of Lassa fever, a zoonotic disease associated with high morbidity and mortality. Clinical presentation is often nonspecific, and hemorrhagic manifestations occur in only a subset of patients, complicating early diagnosis and treatment^1^. Each year, an estimated 100,000–300,000 infections occur across countries such as Nigeria, Guinea, Sierra Leone, and Liberia, resulting in approximately 5000 deaths, with nearly 58 million people considered at risk^2^. More recent estimations based on computational modeling of seroprevalence in human and rodent populations estimate up to 890,000 infections, resulting in up to 18,000 deaths yearly in the West African region^3^. However, reliable surveillance remains a challenge due to underreporting and limited diagnostic capacity in rural areas. Nigeria, which has the most consistent epidemiological tracking, reported over 10,000 suspected cases in 2024, of which 1300 were confirmed by RT-qPCR. Among these, a case fatality rate of 16.3% was recorded. Given its endemicity, high mortality, and lack of licensed vaccines or broadly effective therapeutics, LASV remains a WHO-prioritized pathogen for urgent research and countermeasure development^4^. Despite its public health significance, key aspects of LASV immunopathogenesis remain poorly understood—particularly the mechanisms underlying disease tolerance in its natural reservoir host. To address this, we investigated how reservoir host-derived dendritic cells (DC) and macrophages (MΦs), the primary targets of LASV in vivo, respond to infection compared to immune agonists and other viruses.

The peri-domestic Natal multimammate mouse (*Mastomys natalensis*, NMM) has been identified as the primary natural reservoir and a key source of zoonotic spillover of LASV^5–7^. These rodents asymptomatically harbor LASV, suggesting co-adaptation between the virus and the NMM^8^. In humans, LASV has a cellular tropism for MΦs and DCs, endothelial cells and hepatocytes, all of which highly express the cell surface receptor α-dystroglycan, one of several proposed entry receptors for LASV^9^. In recent years, the establishment and characterization of a breeding colony of NMM has provided the opportunity to study the reservoir-virus relationship in detail^10^. Advances have been made in the development of tools and strategies for the study of NMM immune responses, demonstrating that NMM display a different immune profile than the laboratory mouse (*Mus musculus*)^11^. Also, it has been shown that NMM bone marrow-derived macrophages mount differential interferon (IFN) responses when infected with various mammarenaviruses. Specifically, LASV induces a significantly weaker IFN-mediated response compared to non-pathogenic relatives such as Morogoro virus (MORV) and Mobala virus (MOBV), as evidenced by lower transcriptional induction of canonical interferon-stimulated genes (ISGs) following infection. Furthermore, pretreatment of NMM MΦs with Poly I:C, a synthetic analog of viral double-stranded RNA, was sufficient to restrict LASV replication, supporting the idea that suppression or evasion of IFN signaling is a prerequisite for productive LASV infection in these cells^12^. In in vivo studies, LASV RNA and viral antigen were detected in multiple organs of experimentally infected NMMs, including liver and spleen; however, no associated histopathological lesions or clinical symptoms were observed, consistent with a state of asymptomatic viral persistence^8,13^. These findings suggest a unique host-virus equilibrium in the natural reservoir, where viral replication occurs in the absence of overt pathology or strong antiviral signaling, likely reflecting long-term co-adaptation. In humans, IFN-dependent inhibition of the replication of LASV in MΦs and DCs has been demonstrated^14^. It has also been described that LASV infects and activates human DCs, but impairs their ability to activate co-cultured T cells, whereas the related non-pathogenic Mopeia virus (MOPV) efficiently induces T cell activation, suggestive of a correlation between a dysregulated DC phenotype and the development of disease^15^.

Since MΦs and DCs are primary targets of LASV in humans and non-human primates, we aimed to characterize the response of NMM-derived MΦs and DCs to LASV infection. This allowed us to investigate which immune features may protect the reservoir and pinpoint where virus–host co-adaptations have occurred. We successfully generated DCs and MΦs from the bone marrow of NMM and found that both cell types are permissive to LASV infection. However, LASV failed to induce maturation or activation of these cells, demonstrating that the virus developed specific active mechanisms of immune evasion as a means of co-adaptation in the natural reservoir and that the virus does not elicit an innate immune response in these cells.

## Results

### NMM bone marrow cells differentiate into functionally distinct DCs and MΦs in the presence of recombinant mouse cytokines

**Fig. 1:**
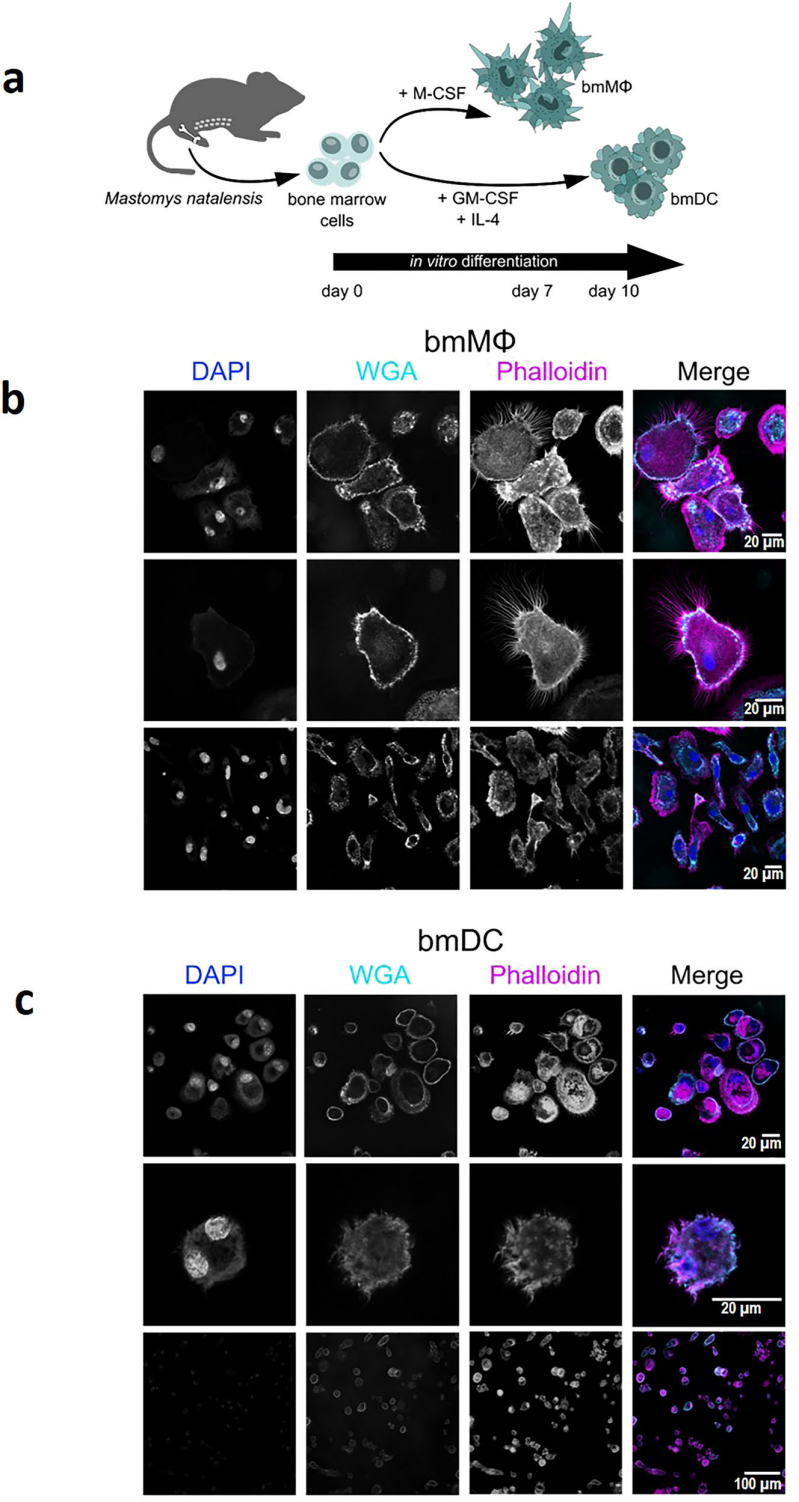
Mouse recombinant cytokines cross-react and induce the differentiation of bone marrow from the Natal multimammate mouse. Schematic representation of the differentiation procedure of bone marrow cells into bmMΦs and bmDCs (**a**). Three independent and representative confocal microscopy fields of bmMΦs (**b**) and bmDCs (**c**) after the differentiation process. Nuclei were stained with DAPI (blue), plasma membranes with WGA (cyan) and phalloidin (magenta). Only the merged images were processed using ImageJ software.

Since DCs and MΦs are dysregulated by LASV in humans and myeloid cells are targets of LASV in the NMM, we sought to generate and characterize MΦs and DCs from NMM bone marrow cells (bmMΦs and bmDCs). We found a high protein sequence similarity of the growth factors needed for in vitro differentiation of bmMΦs and bmDCs (GM-CSF, M-CSF and IL-4) between NMMs and several other rodent species. Hence, we adapted existing protocols and stimulated bone marrow cells with recombinant mouse GM-CSF and IL-4 to generate bmDCs or M-CSF to generate bmMΦs (Fig. 1a; Supplementary Fig. 1a). As expected, bmMΦs were large, elongated, adherent cells, with filopodia extending predominantly from two opposing ends of the cell rather than being evenly distributed around the membrane (Fig. 1b). bmDCs were non-adherent spherical cells, that displayed crown-like structures of dendrites protruding from their surface (Fig. 1c). The morphology of both was also significantly different to that of undifferentiated bone marrow cells, which did not survive culturing in the absence of the cytokines (Supplementary Fig. 1b).

**Fig. 2:**
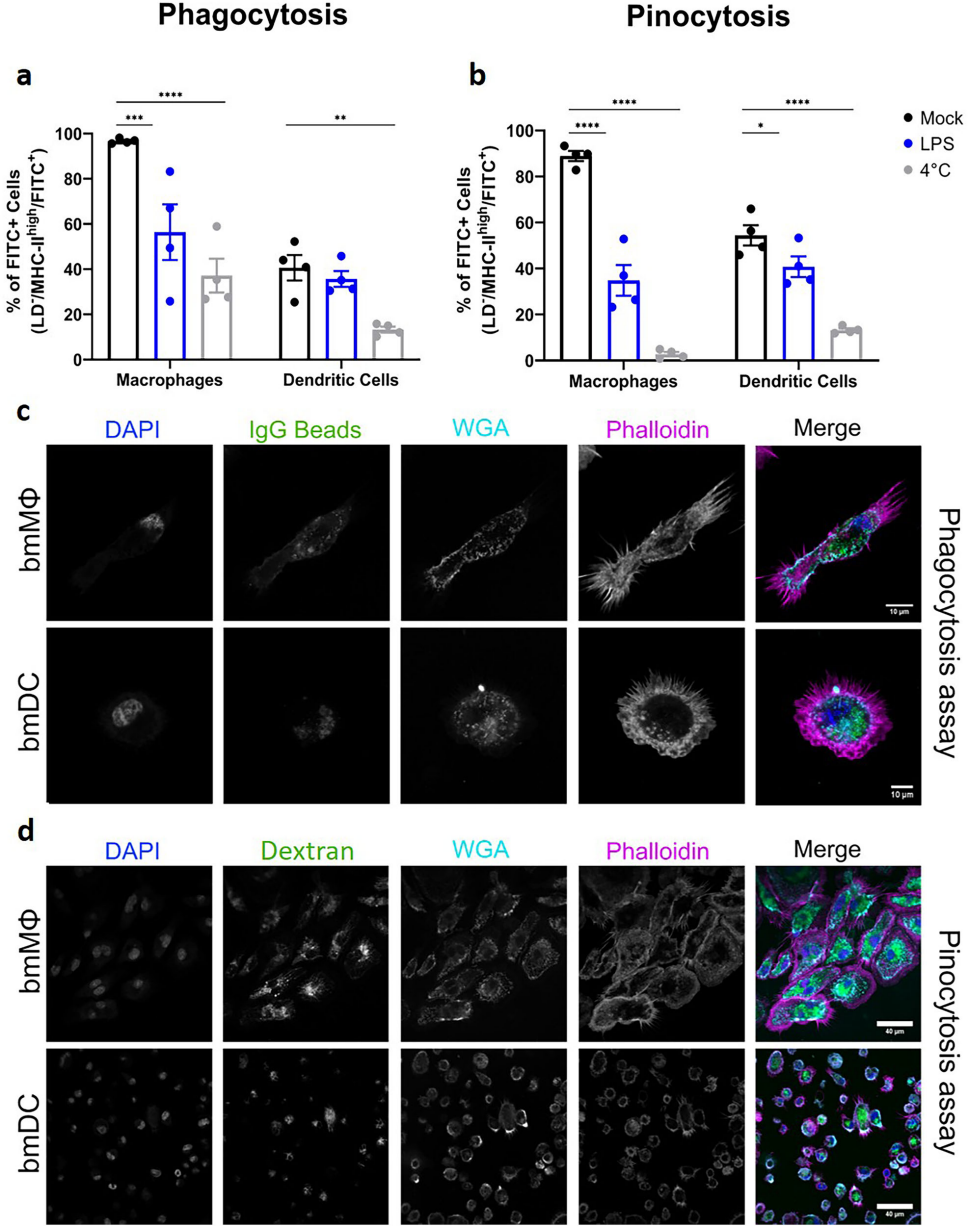
MΦs and DCs display phago- and pinocytic activity and respond to LPS. Flow cytometric assessment of the proportion of positively-labeled bmMΦs and bmDCs after 24 h of stimulation with LPS to evaluate their effect on the phagocytic (**a**) or pinocytic (**b**) activity of these cells. Data shown correspond to cells from four animals. Bars represent the mean ± standard deviation (SD). The data was analyzed using a two-way-ANOVA, with a Dunnett *post-hoc* test for multiple comparisons. ****p < 0.0001; ***p < 0.001; **p < 0.01; and *p < 0.05. Representative confocal microscopy images of bmMΦs and bmDCs after incubation with fluorescently-labeled IgG-beads or dextran for the assessment of the phago- (**c**) or pinocytosis (**d**). Nuclei were stained with DAPI (blue), plasma membranes with WGA (cyan) and phalloidin (magenta). Only the merged images were processed using ImageJ software.

We next examined classical functional features of bmMΦs and bmDCs as professional antigen-presenting cells (APCs) by assessing their capacity for antigen uptake via phagocytosis and pinocytosis. Flow cytometric analysis (gating strategy in Supplementary Fig. 2a–d) confirmed that both cell types expressed MHC-II and efficiently internalized IgG-coated beads (Fig. 2a) and fluorescent dextran (Fig. 2b), demonstrating robust phagocytic and pinocytic activity, respectively. These observations were further supported by confocal microscopy, where both bmMΦs and bmDCs displayed conserved morphology and visible dye incorporation (Fig. 2c, d). Treatment with bacterial lipopolysaccharide (LPS) inhibited both uptake pathways, indicating functional responsiveness to innate immune stimuli. Similarly, assays conducted at 4 °C abolished phagocytosis and pinocytosis (Supplementary Fig. 2), confirming the energy dependence of these active processes. Notably, the extent of LPS-induced inhibition differed between bmMΦs and bmDCs, supporting the notion that these are distinct and functionally specialized myeloid populations. Together, these findings validate the dynamic immune competence of NMM-derived APCs and their suitability for downstream infection and activation studies.

### LASV replicates efficiently in both NMM bmMΦs and bmDCs

**Fig. 3:**
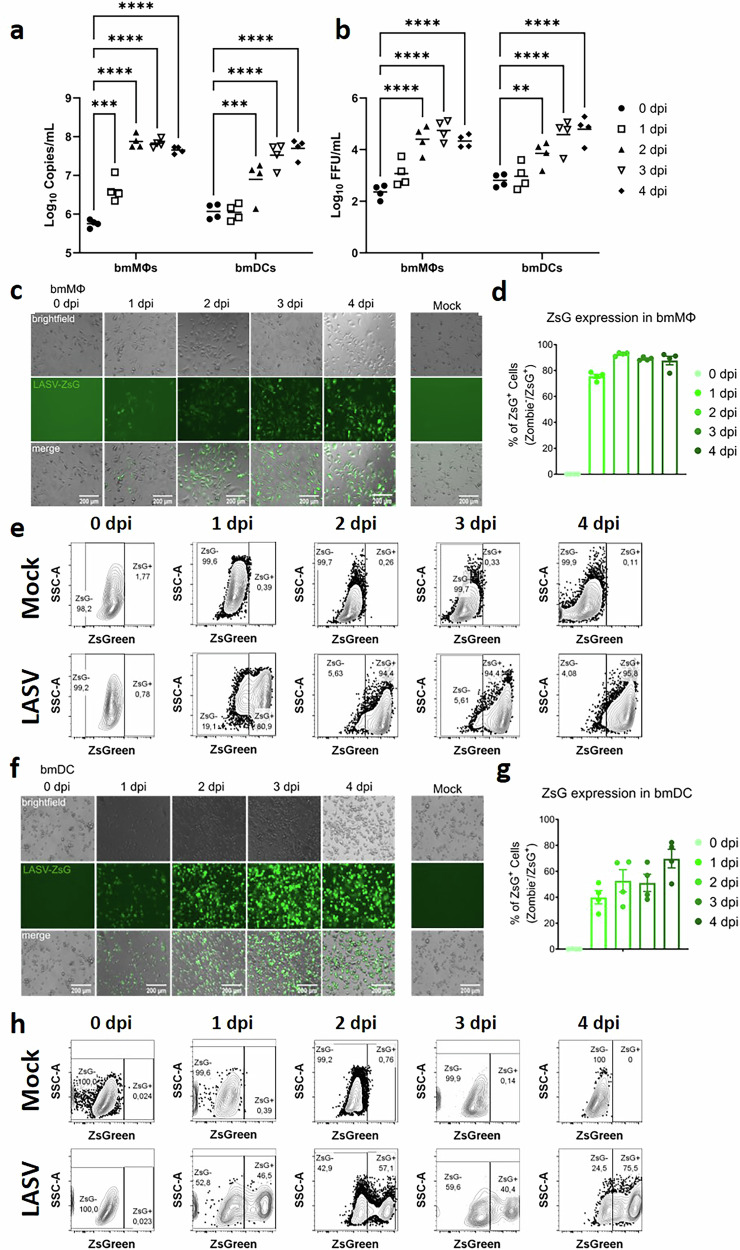
bmMΦs and bmDCs from NMM support Lassa virus infection and viral replication. **a** Viral RNA kinetics in supernatants from bmMΦs and bmDCs infected with LASV (MOI = 0.1), quantified by RT-qPCR at the indicated time points. **b** Infectious virus production measured by focus-forming assay (FFU/mL) from the same supernatants. **c** Representative fluorescence microscopy images of bmMΦs infected with LASV-ZsGreen (MOI = 2). **d** Flow-cytometric quantification of ZsGreen-associated fluorescence in bmMΦs across treatments. **e** Representative flow-cytometry plots for bmMΦs showing ZsGreen fluorescence in LASV-infected versus mock-treated samples, illustrating the gating strategy for ZsG⁺ cells. **f** Representative fluorescence microscopy images of bmDCs infected with LASV-ZsGreen (MOI = 2). **g** Flow-cytometric quantification of ZsGreen-associated fluorescence in bmDCs. **h** Representative flow-cytometry plots for bmDCs showing ZsGreen fluorescence in LASV-infected versus mock-treated samples. Data correspond to four animals. Lines represent geometric means in viral kinetics panels; bars represent arithmetic means in flow-cytometry summaries. Statistical analysis was performed using two-way ANOVAs with Dunnett’s *post-hoc* tests. ****p < 0.0001; ***p < 0.001; **p < 0.01.

To assess the susceptibility of NMM-derived bmMΦs and bmDCs to LASV infection, we infected each cell type at a low multiplicity of infection (MOI = 0.1) and monitored viral replication over time. Viral RNA accumulated in both supernatants from bmMΦs and bmDCs, with higher early replication in bmMΦs and a delayed but steady increases in bmDCs that reached comparable levels by later time points (Fig. 3a). This pattern was confirmed at the level of infectious virus, as fluorescent focus titration of the same supernatants showed a parallel rise in infectious particle production in both cell types (Fig. 3b). Together, these data demonstrate that LASV is able to productively infect and replicate in bmMΦs and bmDCs from the NMM, with no major differences in replication dynamics detectable at the population level under these conditions.

In parallel, we infected bmMΦs and bmDCs at a MOI of 2 with a recombinant LASV expressing a ZsGreen reporter^16^, and observed the expression of virus-associated fluorescence as early as 1 day post-infection (DPI) under a fluorescent microscope (Fig. 3c, f). Quantification of ZsGreen signal as a proxy for the percentage of virus-infected cells by flow-cytometry demonstrated a time-dependent increase in cells expressing ZsGreen, reaching over 80% of bmMΦs by 4 DPI (Fig. 3d, e). A similar pattern was observed for bmDCs, even though ZsGreen-expressing cells only reached approximately 70% by 4 DPI, (Fig. 3g, h).

### LASV infection induces partial maturation in NMM-infected bmDCs but not in bmMΦs

**Fig. 4:**
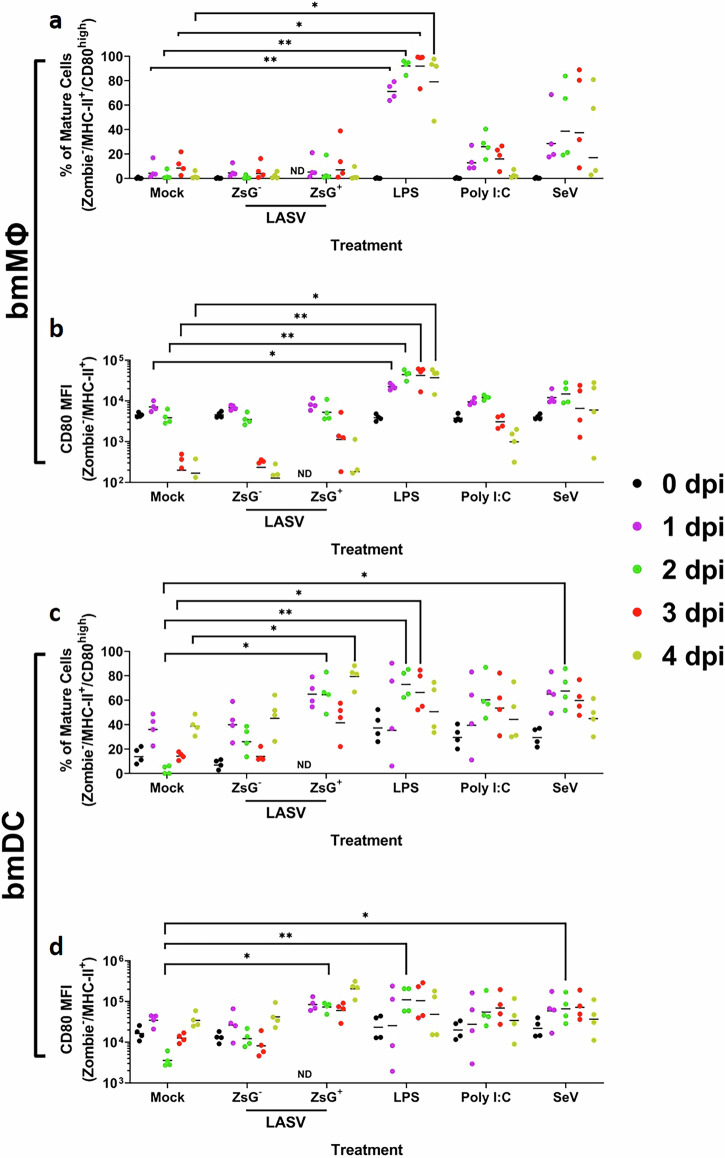
LASV selectively induces CD80 surface upregulation in infected bmDCs. Flow cytometric quantification of the percentage of mature cells (MHC-II⁺/CD80^high^; **a**,**c**) and CD80 mean fluorescence intensity (MFI; **b**,**d**) in bmMΦs (**a**,**b**) and bmDCs; **(c**,**d**) from NMM. Cells were either mock-infected, stimulated with LPS, Poly I:C, SeV, or infected with LASV-ZsGreen (MOI = 2) and measured at 0, 1, 2, 3, and 4 DPI. In LASV-infected groups, ZsG⁺ cells represent productively-infected cells (expressing viral ZsGreen), while ZsG⁻ cells correspond to uninfected bystander cells from the same cultures. Data shown represent cells from four animals. Lines indicate arithmetical means. Statistical analysis was performed using two-way ANOVAs with Dunnett’s *post-hoc* tests. **p < 0.01 & *p < 0.05.

To assess the impact of LASV infection and immune stimulation on cell viability and maturation, we performed longitudinal flow cytometry analysis of CD80 surface expression from 0 to 4 (DPI). CD80 expression was quantified by median fluorescence intensity (MFI) on Zombie⁻/MHC-II⁺ cells, and the percentage of mature cells was defined as the proportion of Zombie⁻/MHC-II⁺/CD80^high^ cells (gating strategies in Supplementary Fig. 3a–c**)**. In bmMΦs, LPS stimulation led to a statistically significant increase in the percentage of mature bmMΦs compared to mock-treated cells on all time points from day 1 through day 4 (Fig. 4a). Similarly, the CD80 MFI was significantly elevated under LPS treatment across the same days (Fig. 4b), demonstrating a consistent maturation response to bacterial agonist stimulation.

In NMM bmDCs, the percentage of mature bmDCs was significantly elevated relative to mock-treated cells only on day 2 post-stimulation with either LPS or SeV, which continued for LPS-treated cells on day 3 (Fig. 4c). CD80 MFI was also significantly higher compared to mock-treated bmDCs under these same conditions on day 2 (Fig. 4d). These findings confirm that both bmMΦs and bmDCs from NMM retain functional responsiveness to classical innate immune stimuli, and that CD80 upregulation occurs in a stimulus- and time-specific manner.

Notably, we observed a transient decrease in CD80 expression in mock-treated cultures at the isolated 2 DPI time point. As this fluctuation was not accompanied by reduced viability and was not reproducible across adjacent time points, we interpret it as a technical rather than biological effect. Importantly, CD80 upregulation in LASV-infected cells remained significant even when using mock values averaged across stable time points (data not shown).

Infection with LASV did not increase CD80 expression in macrophages at any time point, either in terms of fluorescence intensity or the proportion of mature cells. However, a distinct response was observed in bmDCs. At 2 DPI there was a significant increase in in the percentage of CD80^high^-expressing mature cells and in the CD80 MFI, specifically within the infected (ZsGreen-expressing) population (Fig. 4c, d). This effect was absent in the ZsGreen^-^ (bystander) population. Overall, viability remained stable and high across all time points in both bmMΦs and bmDCs infected with LASV (Supplementary Fig. 3d, e, respectively), indicating that the virus does not exert a cytopathic effect under the conditions tested. These results suggest that LASV can partially induce DC maturation in cells it infects, while failing to activate or mature MΦs under the same conditions.

### LASV fails to trigger transcriptional activation of antiviral or maturation-related genes in bmMΦs and bmDCs

**Fig. 5:**
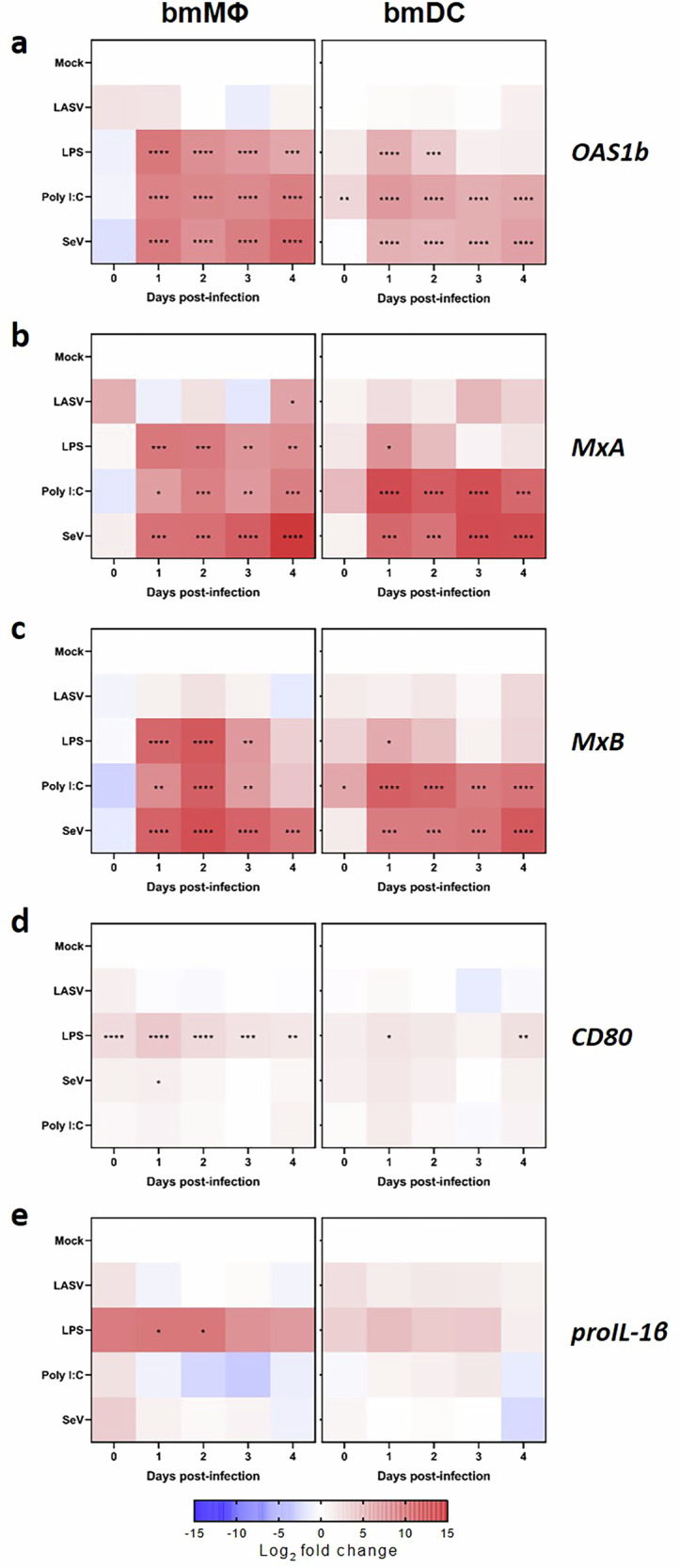
Transcriptomic analysis of selected immune-related genes in bmMΦs and bmDCs after LASV infection or stimulation. Gene expression levels were measured by RT-qPCR in bmMΦs and bmDCs at days 0 to 4 following treatment with mock, LASV, LPS, Poly I:C, or SeV. Heatmaps show the normalized expression values for *OAS1b* (**a**), *MxA* (**b**), *MxB* (**c**), *CD80* (**d**), and *proIL-1β* (**e**). Data shown correspond to cells from four animals. These data were analyzed using a two-way ANOVA, with a Dunnett *post-hoc* test for multiple comparison. ****p < 0.0001; ***p < 0.001; **p < 0.01; and *p < 0.05.

To investigate whether LASV infection induces transcriptional regulation of key antiviral or maturation-related genes in NMM myeloid cells, we profiled the gene expression levels of five representative genes: *OAS1b*, *MxA*, *MxB*, *CD80*, and *proIL-1β* (Fig. 5a–e). Across these genes, LASV consistently failed to induce transcriptional upregulation in both bmMΦs and bmDCs. This was evident even at later time points post-infection, suggesting a broad suppression or evasion of host innate signaling pathways. In stark contrast, the control stimuli (LPS, Poly I:C, and SeV) induced strong gene up-regulation responses across all five genes. Together, these findings confirm that both cell types are functional and competent in mounting canonical antiviral and inflammatory gene responses against common stimulants.

Of particular interest, despite the significant upregulation of surface CD80 protein observed in ZsGreen^+^ bmDCs at 2 DPI (Fig. 4c), CD80 transcript levels remained unchanged in all LASV conditions (Fig. 5d). This discrepancy implies a post-transcriptional mechanism of CD80 protein expression or cell surface trafficking, highlighting a disconnect between cell phenotype and gene expression in LASV-infected cells.

**Fig. 6:**
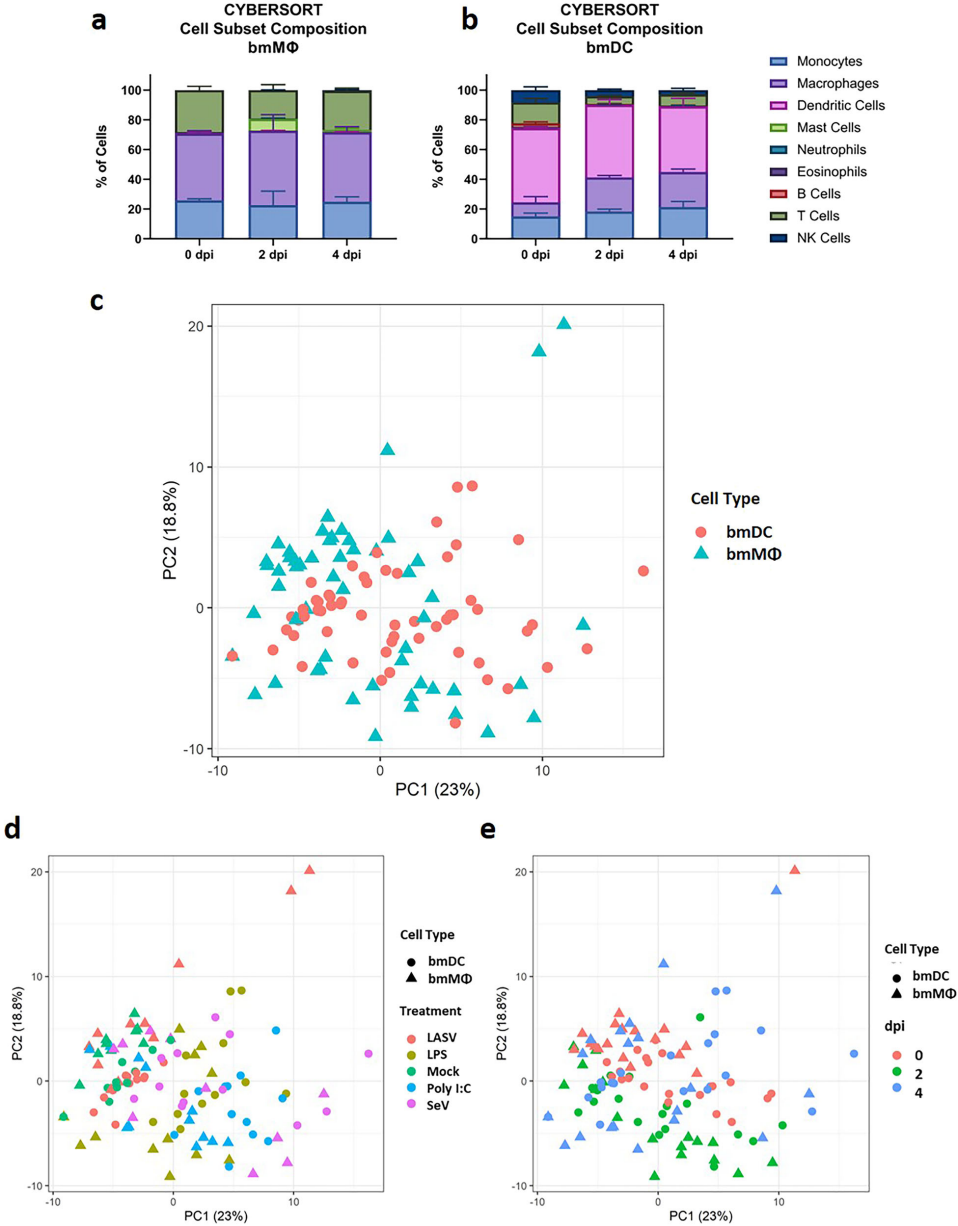
Global transcriptional patterns and cell type stability of NMM-derived APC cultures. CIBERSORTx-estimated immune cell subset composition of mock-treated bmMΦ (**a**) and bmDC (**b**) cultures at 0, 2, and 4 DPI, showing stable proportions over time. Principal component analysis (PCA) of NanoString® expression data from bmMΦs and bmDCs following mock treatment, LASV infection, or stimulation with LPS, Poly I:C, or SeV, colored by (**c**) cell type, (**d**) treatment, or (**e**) day. LASV-infected samples clustered with mock-treated controls, whereas the other stimuli induced distinct, stimulus-specific separation. Each dot corresponds to the average of cells from four animals.

To assess broader transcriptional responses to LASV and control stimuli, we performed targeted transcriptomic profiling of bmMΦs and bmDCs using a custom NanoString® codeset comprising 143 housekeeping and immune-related genes specific for *Mastomys natalensis*. The resulting dataset was first used for CIBERSORTx deconvolution to estimate immune cell subset composition in mock-treated cultures over time. The predicted profiles were dominated by the expected lineage identity, macrophages for bmMΦ cultures and DC for bmDC cultures, alongside a proportion of monocytes, likely reflecting transcriptional overlap with progenitors due to the targeted nature of the codeset. These proportions remained stable from day 0 to day 4, indicating that the differentiated cultures maintained their identity and did not undergo detectable cell loss or de-differentiation during the experimental period (Fig. 6a, b). We then applied principal component analysis (PCA) to the same NanoString® dataset to examine global transcriptional trends. Regardless of grouping by cell type (Fig. 6c), treatment (Fig. 6d), or time point (Fig. 6e), LASV-infected samples did not form distinct clusters from mock-treated cells, indicating that LASV induces minimal transcriptomic perturbation.

**Fig. 7:**
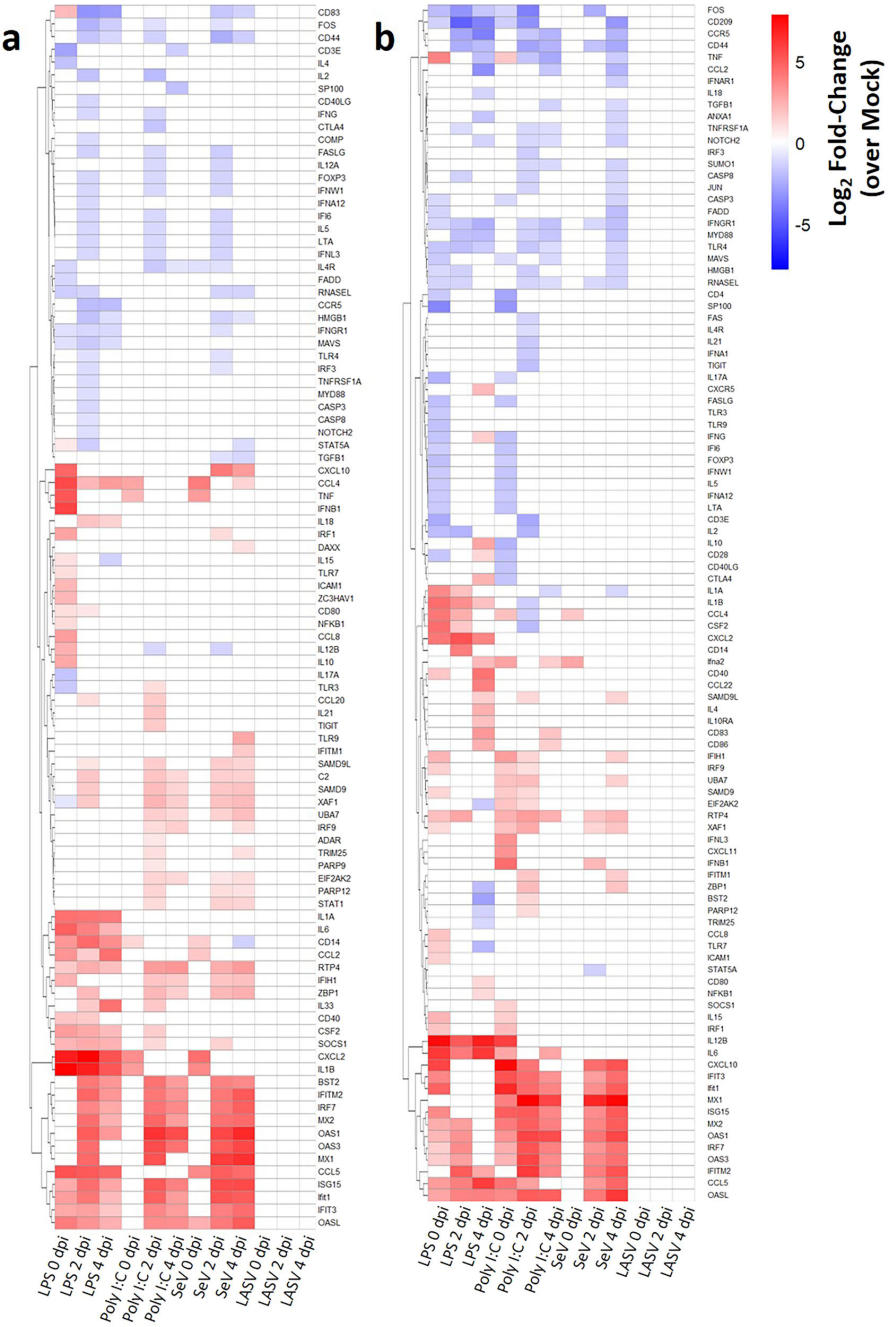
Global transcriptomic profiling of bmMΦs and bmDCs in response to LASV infection or immune stimulation. Expression of 143 immune-related genes was quantified using a custom NanoString® CodeSet in bmMΦs and bmDCs at 2 and 4days post-treatment with mock, LASV, LPS, Poly I:C, or SeV. Differential expression was assessed using a limma–voom workflow with linear modeling and empirical Bayes moderation. Heatmaps display log₂ fold-changes relative to mock-treated controls for (**a**) bmMΦs and (**b**) bmDCs, restricted to genes meeting significance thresholds (FDR < 0.05 and |log₂FC | > 1); non-significant values are masked in white. Columns are ordered by treatment and time point in a predefined canonical layout. Data represent normalized counts from cells from four animals per group.

We next performed a comprehensive differential expression analysis using a limma-voom^17^ workflow to assess transcriptional responses of NMM-derived bmMΦs and bmDCs to LASV infection and to three classical immune stimuli. Consistent with our previous observations, LASV infection failed to induce significant transcriptional changes in either cell type at any time point, with no differentially expressed genes (DEGs) detected after false discovery rate (FDR) correction (Fig. 7). This absence of LASV-driven responses was not attributable to technical limitations, as the raw fold-change distributions demonstrated robust dynamic ranges across the entire gene panel for all conditions (Supplementary Fig. 4).

In contrast, all control stimuli elicited strong and stimulus-appropriate transcriptional signatures in both bmMΦs and bmDCs, confirming the responsiveness and functional identity of the differentiated APCs. In bmMΦs, LPS induced an early, strong, and sustained activation profile, with canonical genes such as *CXCL2*, *proIL1α*, *proIL1β*, *CD14* and *IL6* showing ~5-log₂-fold increases already at 0 DPI (1 HPI), which remained comparatively stable through 4 DPI. Poly I:C stimulation also triggered a pronounced antiviral program, particularly at 2 and 4 DPI, characterized by upregulation of *TLR3*, *OAS1*, *IFIH1* and *ADAR*. SeV infection produced a pattern broadly similar to Poly I:C, but with especially strong induction of *Mx1* and *CCL5*.

bmDCs also mounted robust and stimulus-specific transcriptional responses, though with distinct kinetics compared to macrophages. LPS-treated bmDCs showed rapid induction of inflammatory mediators, including *CCL4*, *CXCL2*, *IL6* and *IL12β*; with particularly dynamic modulation of *TNF*, which was upregulated at 0 DPI, absent as a DEG at 2 DPI, and then strongly downregulated by 4 DPI. Poly I:C stimulation in bmDCs produced a biphasic response: a group of antiviral genes (*IFIH1*, *IRF9*, *RTP4*) peaked early and then declined, whereas a second cluster (*CXCL10*, *IFIT3*, *Mx1*, *Mx2*) remained strongly induced across time points. SeV treatment yielded a response highly similar to Poly I:C but delayed, with few early DEGs at 0 DPI and robust ISG induction emerging at 2 DPI and persisting through 4 DPI.

Together, these results demonstrate that both NMM-derived bmMΦs and bmDCs exhibit canonical, stimulus-appropriate transcriptional activation but retain distinct cell type–specific response profiles,

supporting the fidelity of our differentiation protocol. Importantly, the complete absence of LASV-induced DEGs strongly reinforces the conclusion that LASV does not trigger detectable innate transcriptional activation in these reservoir-derived myeloid cells.

## Discussion

Understanding how natural reservoirs interact with the zoonotic viruses they carry is fundamental to identifying key immune correlates of disease versus tolerance. In the case of viral hemorrhagic fevers (VHFs), these questions are particularly relevant, as many of these viruses (including *arenaviruses* and *hantaviruses*) are maintained in asymptomatic reservoir hosts despite ausing high mortality rates in humans. It remains unclear which immune responses are conserved across species and which are uniquely dysregulated in disease-prone hosts. LASV infects myeloid cells in both humans and its natural reservoir, NMMs. While previous work has characterized the transcriptional response of NMM-derived bmMΦs to *Mammarenavirus* infection, notably demonstrating a weaker IFN response to LASV compared to MORV or MOBV^12^, our study extends these findings by including bmDCs, assessing both phenotypic and functional maturation, and applying a broader transcriptomic approach. The combination of cell-type comparison, functional validation, and multi-day profiling offers a more comprehensive view of APC responses to LASV in its natural reservoir in vitro, and provides novel insights into virus-specific modulation of innate immunity.

In contrast to the absence of transcriptional activity in response to LASV infection, all three control stimuli elicited strong and stimulus-specific gene expression programs in both bmMΦs and bmDCs. LPS induced a rapid and sustained inflammatory signature in both cell types, whereas Poly I:C and SeV triggered robust antiviral transcriptional responses with kinetics characteristic of TLR3- and RIG-I–mediated sensing, respectively. Although PCA based on condition-averaged profiles revealed some dispersion along PC1, this pattern reflects the inherent inter-individual variability of ex vivo cells from an outbred colony rather than stimulus-specific transcriptional programs, consistent with the absence of LASV-induced DEGs. While the exact timing and amplitude of individual genes differed between bmMΦs and bmDCs, the overall patterns were highly consistent with the expected biology of each stimulus and with the lineage-specific functional roles of macrophages and DCs. These canonical and distinct activation profiles confirm that the differentiated APCs were immunologically competent and responded as anticipated, thereby validating the robustness of our differentiation protocol and transcriptomic workflow. The ability of the differentiated cells to phagocytose and perform pinocytosis, and their classical morphology and surface expression profiles, including MHC-II and CD80, further support their identity as functional APCs^18–23^. All experiments were performed using male NMMs, and while sex-dependent immune differences cannot be entirely excluded, most reported effects in immunology are mediated by circulating sex hormones^24^, which are absent in our ex vivo culture system, minimizing the likelihood of major sex bias in the responses observed here.

Interestingly, the only detectable response to LASV infection was a modest but significant increase in surface CD80 expression on bmDCs at 2 DPI. This occurred without any accompanying upregulation at the transcript level. No such change was observed in bmMΦs. This surface-specific effect suggests LASV may subtly modulate dendritic cell phenotype without triggering broader immune activation. Since only a limited set of cross-reactive antibodies is currently available for NMM myeloid cells, MHC-II was used as a lineage-defining marker for both bmMΦs and bmDCs. For this reason, MHC-II MFI could not be meaningfully evaluated as an activation readout, as the marker itself forms the basis of the gating strategy used to identify the APC populations. Thus, we analyzed the maturation of the APCs by relying on the CD80 surface expression.

Human DCs infected with LASV upregulate maturation markers, but often fail to present antigen effectively or activate T cells^15^. This failure is considered a key contributor to immune dysfunction during infection. However, our results in NMM cells reveal that a similar suppression of innate responses can occur in the absence of disease, challenging the notion that impaired myeloid activation alone drives pathology. Instead, the downstream immune context, particularly the coordination with adaptive responses, may determine disease outcome.

In humans, LASV possesses potent innate immune evasion mechanisms that likely contribute to this early silencing. The LASV-NP exoribonuclease degrades immunostimulatory dsRNA and blocks IRF3/IRF7 activation, while the LASV-Z protein inhibits RIG-I/MAVS signaling and limits type I IFN induction^25^. Together, these mechanisms can contribute to the suppression of APC activation. Our findings suggest that, if present in NMMs, this early suppression is tolerated without harmful consequences, whereas in humans it may synergize with dysregulated adaptive immunity to promote disease.

LASV targets tissue-resident monocytes and MΦs in vivo in humanized mouse models, where their activation has been linked to disease severity in a T cell-dependent manner^26^. However, here we show that NMM-derived bmMΦs can be infected in the absence of any signs of inflammatory response. Recent in vivo studies have shown that adult NMMs clear LASV infection, whereas neonatal animals support persistence, likely due to the incomplete development or functional limitations of their T cell responses^13^. This supports a model in which early immune evasion allows for viral seeding, but clearance depends on a competent adaptive response.

This model is consistent with what has been described for human monocyte-derived MΦs (moMΦs), which are also permissive to LASV infection but often fail to mount effective antiviral responses^14^. Various mechanisms of immune evasion have been attributed to LASV, including suppression of RIG-I signaling^27^ and inhibition of type I IFN pathways^28^. However, our findings show that these strategies are not exclusive to human hosts, and may also be part of the virus’s adaptation to its natural reservoir. This suggests that immune evasion is not inherently the cause of pathogenesis, but becomes harmful when the immune system fails to compensate appropriately.

Different natural reservoir species appear to have evolved divergent strategies to manage virus-host balance. Deer mice infected with Sin Nombre virus develop a regulatory T cell–mediated tolerance that permits lifelong infection and shedding, without pathology^29^. Egyptian rousette bats infected with Marburg virus mount a limited but well-balanced proinflammatory response that contributes to viral clearance in the absence of disease^30^. These examples emphasize that disease tolerance can emerge from immune suppression or immune activation, depending on host–pathogen co-evolution. Moreover, in the mouse lymphocytic choriomeningitis virus (LCMV) system (another rodent-borne old-world mammarenavirus), early activation of DCs and MΦs enables strong T cell priming that shapes both pathology and viral control. After intracerebral infection, mature APCs efficiently activate CD8⁺ T cells, driving fatal choriomeningitis, while CD4⁺ T cells mediate a slower, non-fatal form. CD8⁺ T cells are also essential for systemic clearance, as mice lacking CD8⁺ cells retain high viral loads long after wild-type animals have resolved infection^31^. Likewise, the distinction between acute and chronic Cl13 infections depends on APC-supported T cell and Bcell responses, including the delayed TFH-driven development of neutralizing and non-neutralizing antibodies during chronic infection^32^.

In contrast, our data show that LASV infection in NMM is not associated with overt activation of bmMΦs or bmDCs under the conditions examined, indicating that these cells may not undergo robust early activation in this experimental setting. Nevertheless, adult NMM are known to clear LASV and mount virus-specific antibody responses^8,13^, implying that effective antiviral immunity is ultimately established. This raises the possibility that immune activation in the reservoir host may involve alternative antigen-presenting or innate immune populations, such as pDCs, NK cells, or tissue-resident MΦs, including Kupffer cells, and suggests that the hierarchy of APC involvement during LASV infection may differ from that described in the LCMV mouse model.

In our study, the lack of transcriptional responses to LASV infection was striking. Across both targeted qPCR panels and broader NanoString® analyses, neither IFN-related genes nor costimulatory markers were upregulated in response to LASV. In bmDCs, surface CD80 expression increased progressively over the 1–4 DPI time course, reaching statistical significance at 2 and 4 DPI, whereas no CD80 upregulation was detected in bmMΦs. This suggests that innate immune recognition of LASV is either actively suppressed or fundamentally absent in NMM-derived APCs. The contrast with strong responses to LPS, Poly I:C, and SeV confirms that this is not due to a general dysfunction of the cells, but rather to a highly selective suppression by LASV. The surface-specific upregulation of CD80, despite no transcriptional induction, suggests a post-transcriptional mechanism. This could resemble the regulated recycling and membrane translocation seen in other immune and metabolic pathways, such as MHC-II^33^ or the widely studied GLUT4^34^ trafficking, where surface expression increases without corresponding changes in gene transcription or total protein levels. A similar mechanism could explain the isolated CD80 surface increase in LASV-infected bmDCs. Notably, the same LASV stock used in this study has been employed in parallel experiments within our laboratory, where it triggered transcriptional activation of unfolded protein response (UPR) and integrated stress response (ISR) pathways in human hepatocytes^35^, supporting its retained infectivity and capacity to engage host cellular pathways under appropriate conditions.

We acknowledge that our study is limited to bone marrow–derived APCs, and future work using directly isolated tissue-resident populations will be important to fully assess LASV immune evasion in vivo. Ongoing efforts in our group to develop and refine such primary-cell isolation tools may help address this limitation in subsequent studies^36^. In addition, our sampling strategy did not include intermediate early time points between 1 hpi and 24 HPI, which may restrict our ability to detect very rapid innate sensing events; future studies incorporating finer temporal resolution will be valuable to fully capture these early dynamics.

Together, our findings support a model in which LASV avoids myeloid cell activation to facilitate early infection in the NMM. In adult NMM, the virus could be eventually cleared through T cell–mediated mechanisms, but in neonates (where T cell function is limited) LASV can persist. This fits with the observation that viral clearance in the NMM is age-dependent and that persistent infection occurs when innate suppression is not counterbalanced by adaptive immunity^13^. In humans, where immune coordination can break down or become hyperactivated, the same innate suppression may contribute to pathology rather than tolerance.

In conclusion, our study provides new insight into the immunobiology of LASV in its natural host. The data suggest that LASV induces little to no immune activation in myeloid cells from the NMM, except for a limited surface response in DCs. These findings contribute to our understanding of host-pathogen coadaptation and raise new questions about what drives disease in humans. Comparative models such as this are essential for identifying which immune features correlate with disease, and which support persistence or clearance without harm. Future work comparing LASV to non-pathogenic *mammarenaviruses* like MOPV in both humans and natural hosts may help reveal the immune checkpoints that determine the outcome of infection.

## Methods

### Biosafety

All experiments with Risk Group 4 viruses were conducted under maximum containment conditions in the biosafety level 4 (BSL4) laboratory of the Robert Koch Institute, in accordance with standard operating procedures institutionally approved by the Robert Koch Institute.

### Ethics Statement

Multimammate mice’s samples were obtained according to the procedures approved by ethics committee of the Berlin state’s office for Health and Social (LAGeSO Berlin) and the Robert-Koch Institute (agreement TN0001/22).

### Virus

LASV strain Josiah (GenBank Accession: NC_004296.1, NC_004297.1) is a human isolate^37^ and was kindly provided by Dr. Feldmann from Rocky Mountains Laboratory. Recombinant LASV encoding the ZsGreen reporter gene was generated and kindly provided by Dr. Albariño from the Center of Diseases Control^16^. Both were propagated on Vero76 cells (ATCC #CRL-1587) using DMEM (Sigma) supplemented with 100 U/mL penicillin, 100 µg/mL streptomycin (Gibco), 2 mM L-glutamine, (Gibco) and 2% FCS (Biochrom).

Sendai virus strain Cantell (SeV, ATCC VR-907 Cantell) was obtained from Charles River Laboratories (Wilmington, MA).

### Generation of bone marrow-derived MΦs and DCs

A MNN (*Mastomys natalensis*) outbred colony, descending from wild-caught animals from Mali^10^, has been bread and maintained at the animal facility of the Robert Koch Institute. Four male adult multimammate mice between 16 and 20 weeks old were euthanized by isofluorane overdose, cervical dislocation and exsanguination via cardiac puncture according to the approved protocol.

Femurs and tibias were exised and rinsed in 70% ethanol and then in PBS. After that, the epiphyses of the bones were cut and the bone marrow was extracted by perfusing the bones with RPMI 1640 (Sigma) supplemented with 100 U/mL penicillin, 100 µg/mL streptomycin (Gibco), 2 mM L-glutamine (Gibco), 1% HEPES (Gibco) and 10% FCS (Biochrom). Remaining erythrocytes were lysed by washing the cells with ACK (Gibco) and then the cells were passed through a 70 µm cell strainer (Miltenyi Biotec) to eliminate bone or tissue debris.

For the differentiation of bmMΦs, cells were cultured for 7 days in the RPMI media, supplemented with 25 ng/mL of mouse recombinant M-CSF (R&D Systems) at 37 °C and 5% CO_2_. On day 3, the same volume of fresh medium was added.

For the differentiation of bmDCs, cells were cultured for 10 days in the RPMI media, supplemented with 25 ng/mL of mouse recombinant GM-CSF (R&D Systems) and 25 ng/mL of mouse recombinant IL-4 (R&D Systems) at 37 °C and 5% CO_2_. On day 3, the same volume of fresh medium was added and on day 7, 50% of medium was removed and replaced with fresh medium.

### Confocal Microscopy

For confocal fluorescence microscopy imaging, cells were stained with wheat germ agglutinin (WGA)-AleaFluor594 (Invitrogen), fixed with 10% formalin (HistoFix, Roth) and then stained with Acti-Stain 670 (Cytoskeleton) and DAPI (RotiMount, Roth). All according to manufacturer’s instructions. Imaging was performed using the Stellaris 8 confocal microscope (LEICA) from the unit for Advanced Light and Electron Microscopy at the Center for Biological Threats and Special Pathogens from the Robert Koch Institute. Image processing was performed using ImageJ software^38^.

### Phagocytosis and Pinocytosis Assays

Differentiated cells were stimulated with 2 µg/mL LPS (InvivoGen, #tlrl-eblps, standard) for 21 h prior to the assay. Phagocytosis analysis with FITC-beads was performed according to manufacturer instructions (Cayman-Chem). After 2 h of incubation at 37 °C with FITC-IgG-beads, the cells were kept permanently on ice to be microscopically or flow cytometrically analyzed. Signals of extracellular non-internalized beads were quenched by addition of Trypan blue (0.04%, Lonza) for 2–3 min and two subsequent washes with cold PBS prior to measurements. Fixable Live/Dead Yellow (Invitrogen) and antibody staining (MHC-II) were performed afterwards, for flow cytometric analysis.

Pinocytosis was measured as the intracellular absorption of soluble FITC-Dextran (Sigma-Aldrich; 40,000 Da, final conc. 0.5 mg/mL) after 2 h of incubation at 37 °C. Free unbound FITC-Dextrane was removed by two washes with ice-cold PBS prior to measurements, and stainings were performed as described above.

### Stimulation and Infection

On the corresponding days, bmDCs or bmMΦs were infected with a MOI of 0.1 or 2 of LASV *wild-type* or LASV-ZsGreen for 1 h at 37 °C. After that, the supernatant was removed and fresh medium was added to the cells for the corresponding time. Epifluorescence microscopy pictures were taken for the ZsGreen samples prior to harvesting. In parallel, the cells were stimulated with 8 µg/mL of LPS (InvivoGen), 25 ng/mL of Poly I:C (HMW, InvivoGen) or 30 HAU of SeV, for the corresponding timepoints.

### Virus Quantitation

For viral quantification, supernatants were collected and titrated over Vero 76 cells. After 1 h of infection, overlay medium (previously described DMEM + 1.5% carboxymethilcellulose) was added and cells were incubated for 5 days at 37 °C. After that, overlay medium was removed and the cells were fixated overnight with 10% formalin (HistoFix, Roth) for the inactivation of the virus. For the ZsGreen virus, the fluorescent focus units were directly counted on a fluorescence microscope. For the *wild-type* virus, the cells washed with water and incubated with permeabilization buffer (PBS 0.5% Triton X-100) for 30 min and then incubated with blocking buffer (PBS 2% FCS) for 30 min at room temperature. After that, a primary anti-LASV NP antibody (ProGen #691652) at a 1:1000 dilution overnight at 4 °C and finally an anti-mouse IgG HRP conjugated antibody (Jackson Lab.) at a 1:1000 dilution for 60 min at room temperature. Lastly, the samples were stained using the TBM buffer (RecomBlot, Mikrogen) following the manufacturer’s instructions. Foci were then counted under a lighting plate (Roth).

For viral RNA quantification, supernatants were inactivated using AVL buffer (Qiagen) and 70% ethanol, and then extracted using the QIAamp Viral RNA kit (Qiagen). The quantitative RT-PCR was performed using the primer-probe sets and protocol previously described^8^.

### Cellular RNA extraction and qPCR

Cellular RNA samples were inactivated using RLT buffer with 1% β-mercaptoethanol, and then extracted using the RNeasy kit (Qiagen) following the manufacturer’s instructions.

The RT-qPCR was done using previously reported protocol for *60S, OAS1b, MxA* and *MxB* mRNAs^12^. For *CD80* the forward primer 5’-TTGGCTTCTACGTCGCTCTT-3’, reverse primer 5’-GGATCCTGGGAAACTGTCGT-3’ and detection probe 5’-6-FAM-ACATGCTTCGCTTCCGGGGGTT—BBQ-3’ were used. For *proIL1β* the forward primer 5’-CAAAATACCCGTGGCCTTGG-3’, reverse primer 5’-TGCTTGGGATCCACACTCTC-3’ and detection probe 5’-6-FAM-ACGGCACACCCACACTACAGCT-BBQ-3’ were used. The thermocycler program was the same previously reported for all genes. For the quantification of intracellular viral RNA, same protocol was used as for the supernatant after the corresponding RNA extraction.

### Flow Cytometry

For flow cytometric analysis, the cells were harvested at the corresponding timepoints and first incubated with Fc-block buffer (BioRad #MCA2305) for 15 min. After that, the cells were incubated with MHC-II (Invitrogen #12-5980-82, 1:200), CD80 (Biolegend #104713, 1:200) and ZombieNIR (Biolegend #423106, 1:200) for 15 min at room temperature in the dark. The samples were washed three times with FACS buffer (PBS 1% BSA, 0.1% NaN_3_) and then fixed overnight with 10% formalin (HistoFix, Roth). After that, the cells were centrifuged at 1000 x g for 10 min, and resuspended in FACS buffer. After that, the cells were directly analyzed.

All flow cytometric measurements were performed on a Cytoflex S (Beckmann Colter). The data was graphed and analyzed using FlowJo™ v10.8 Software (BD Life Sciences), using single color and fluorescence-minus-one controls for compensation, and isotype controls for thresholding, and GraphPad Prism v10 for Windows (Boston, Massachusetts USA) software.

### NanoString ® mRNA Analysis

To enable probe design for Mastomys natalensis, a preliminary gene annotation was generated using TOGA (https://github.com/hillerlab/TOGA)^39^. Briefly, pairwise genome alignment chains were computed between human (hg38 assembly) as the reference and *M. natalensis* as the query, using lastz (parameters K = 2400, L = 3000, Y = 9400, H = 2000, default scoring matrix), axtChain (default parameters except linearGap = loose), RepeatFiller, and chainCleaner (default parameters except minBrokenChainScore = 75,000 and -doPairs)^40–42^. TOGA was run with the human GENCODE V38 annotation, and orthologous loci were inferred and classified based on machine learning models incorporating alignments of intronic and intergenic regions. From this preliminary annotation, 143 genes of interest—comprising immune-related targets and reference genes—were selected and submitted to NanoString Technologies Inc., where a custom CodeSet was designed. RNA samples (7 µL per reaction) were hybridized using this CodeSet together with CodeSetPlus reagents and processed on the NanoString SPRINT platform according to the manufacturer’s instructions. Raw counts were normalized using nSolver v4.0 software (NanoString Technologies Inc.) prior to export.

### CIBERSORTx Analysis

The exported normalized counts were analyzed using RStudio. In brief, the data were divided by cell type and by time point. After that, genes with normalized counts lower than 20 were filtered out using the d*yplr* package. The web-based software analysis tool CIBERSORTx was used to estimate the immune cell composition of the in vitro differentiated NMM-derived bmMΦ and bmDC cultures^43^. The data input used the average normalized gene expression counts for all genes in the custom Mastomys natalensis probe CodeSet from mock-infected bmMΦ and bmDC samples. For this study, 100 permutations were performed using a previously published Mus musculus-specific signature matrix^44^.

### Differential expression and clustering analyses

Normalized NanoString counts were analyzed in R (v4.3.1) using the limma and edgeR packages with the voom transformation. For each cell type and day post-infection, differential expression was computed relative to mock-infected controls, applying Benjamini–Hochberg false discovery rate (FDR) correction. Genes with |log₂FC | > 1 and FDR < 0.05 were considered significantly regulated. Hierarchical clustering of significant genes was performed using Euclidean distance and complete linkage, and visualized as heatmaps with the pheatmap package. Supplementary heatmaps included all genes meeting raw *p* < 0.05 or showing any measurable log₂FC across contrasts. Principal component analysis (PCA) was conducted on log₂-transformed normalized expression values using the stats package, and visualized with ggplot2 to assess global transcriptional separation by cell type, treatment, and time point^17^.

## Data availiability

Raw data will be available upon request to the corresponding author.

## Code availiability

All code used in for this manuscript will be available upon request to the corresponding author.

## Supplementary information

Supplementary Material

## Data Availability

Raw data will be available upon request to the corresponding author.
