# Supplementary material for: Lassa virus circumvents macrophage and dendritic cell antiviral defences in its natural reservoir, the Natal multimammate mouse (Mastomys natalensis)
Source: Npj Viruses. 2026 Feb 9;4:9. doi: 10.1038/s44298-026-00177-6 (PMC12886775; doi:10.1038/s44298-026-00177-6)
Supplement: Supplementary file 1 — Supplementary Material [file 44298_2026_177_MOESM1_ESM.pdf]

Supplementary Material

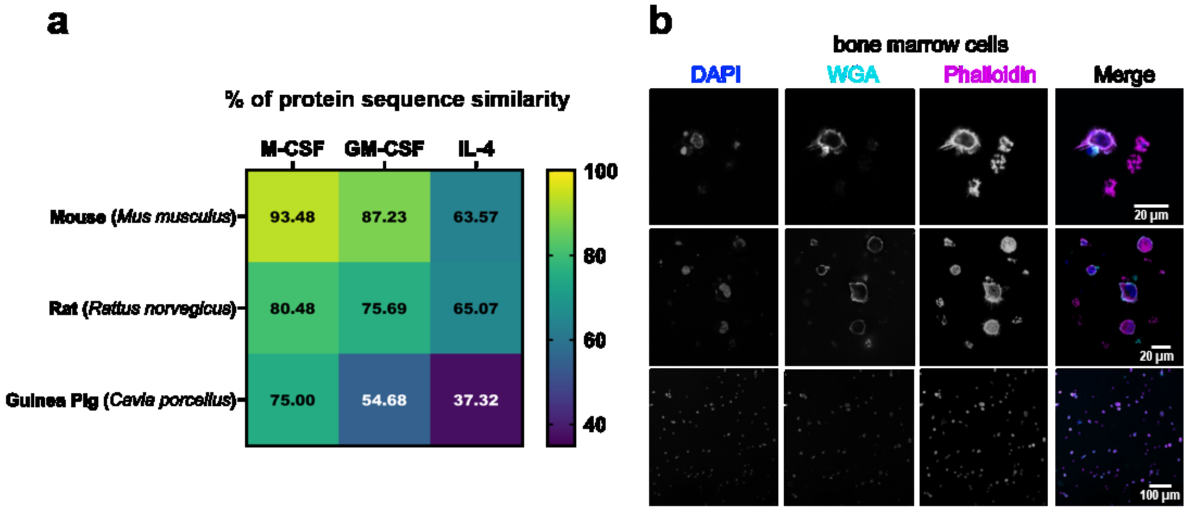

Supplementary Figure 1. Mouse recombinant cytokines induce the differentiation of bone-marrow cells from the natal multimammate mouse. Protein BLAST sequence comparison among highly-studied organisms using Mastomys natalensis as a reference, for the used cytokines (a). Representative confocal microscopy of undifferentiated bone-marrow cells from the natal multimammate mouse (b). Nuclei were stained with DAPI (blue), plasmatic membrane with WGA (cyan) and phalloidin (magenta). Only the merged images were processed using ImageJ software.

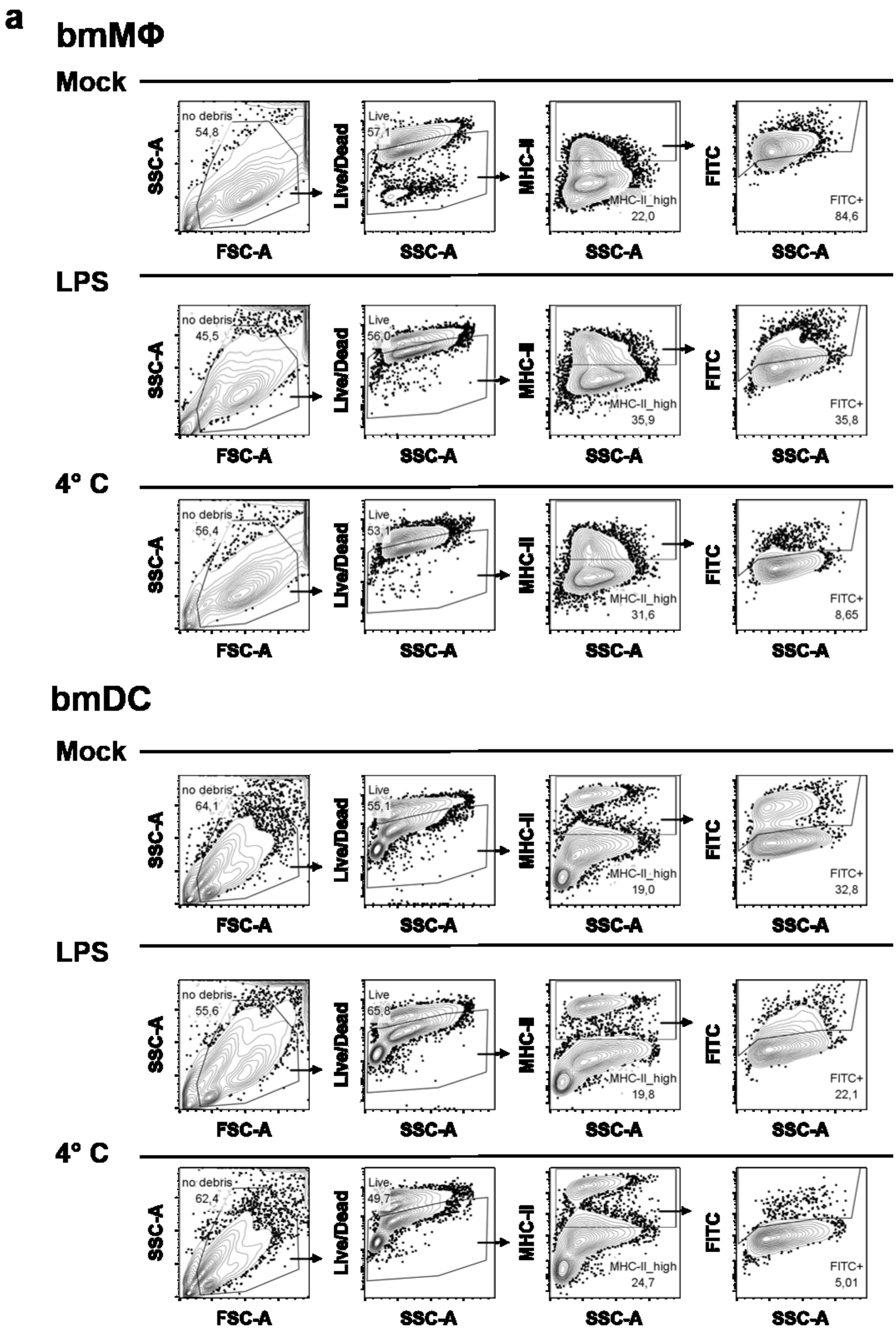
a
bmMΦ
Mock
no debris 54,8
Live 57,1
MHC-II_high 22,0
FITC+ 84,6
LPS
no debris 45,5
Live 56,0
MHC-II_high 35,9
FITC+ 35,8
4° C
no debris 56,4
Live 53,1
MHC-II_high 31,6
FITC+ 8,65
bmDC
Mock
no debris 64,1
Live 55,1
MHC-II_high 19,0
FITC+ 32,8
LPS
no debris 55,6
Live 65,8
MHC-II_high 19,8
FITC+ 22,1
4° C
no debris 62,4
Live 49,7
MHC-II_high 24,7
FITC+ 5,01
SSC-A
FSC-A
Live/Dead
MHC-II
FITC

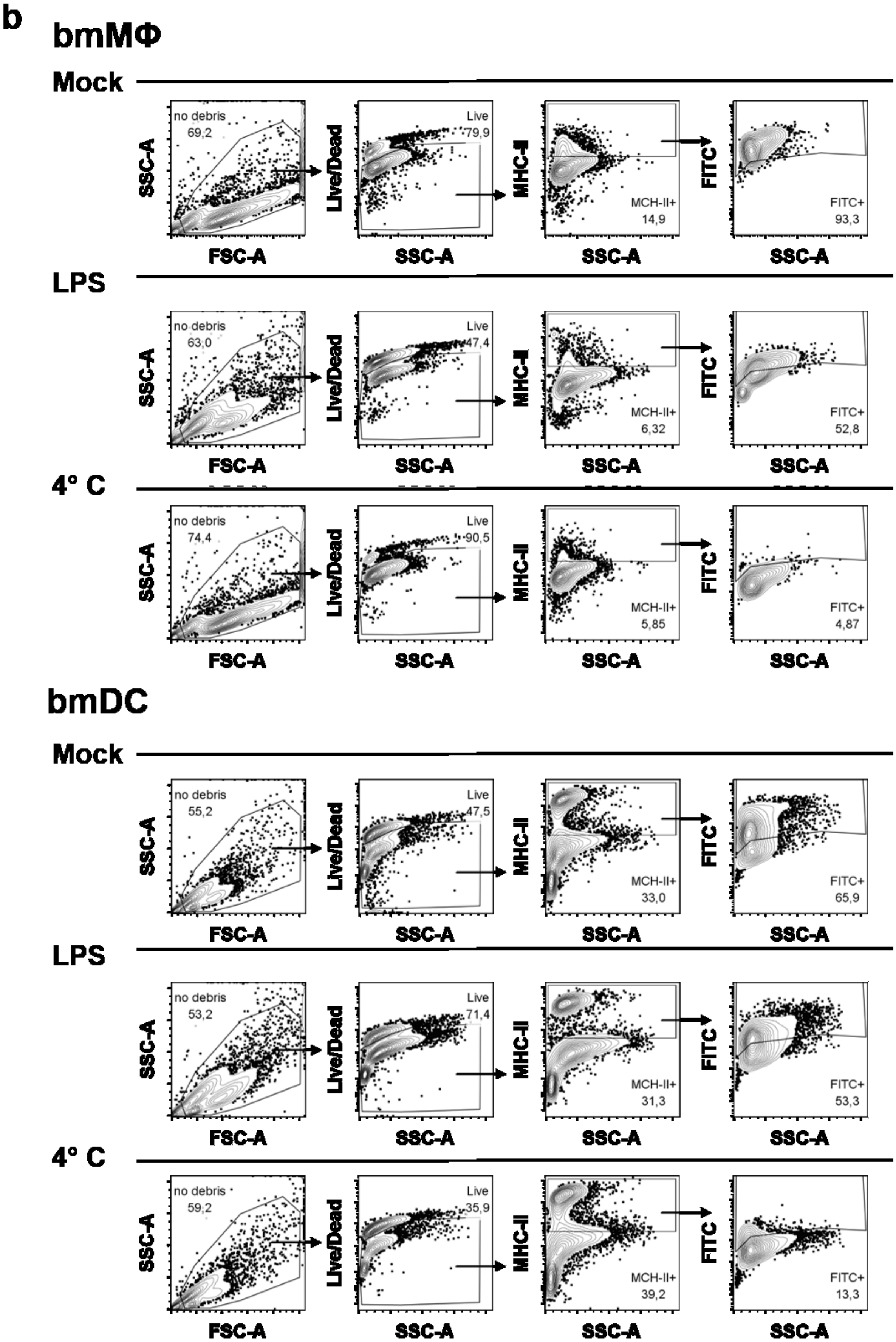
b
bmMΦ
Mock
no debris
69,2
Live
79,9
MCH-II+
14,9
FITC+
93,3
LPS
no debris
63,0
Live
47,4
MCH-II+
6,32
FITC+
52,8
4° C
no debris
74,4
Live
90,5
MCH-II+
5,85
FITC+
4,87
bmDC
Mock
no debris
55,2
Live
47,5
MCH-II+
33,0
FITC+
65,9
LPS
no debris
53,2
Live
71,4
MCH-II+
31,3
FITC+
53,3
4° C
no debris
59,2
Live
35,9
MCH-II+
39,2
FITC+
13,3
SSC-A
FSC-A
Live/Dead
MHC-II
FITC

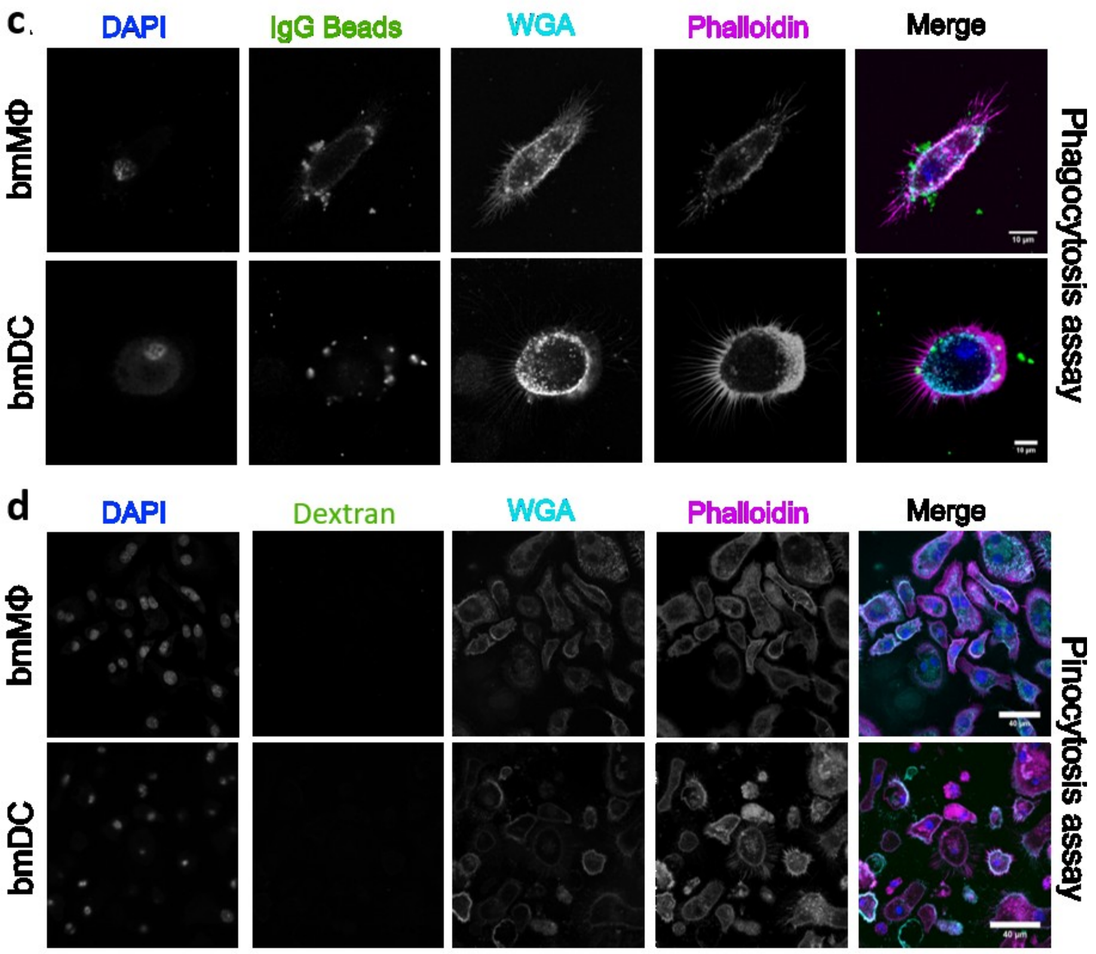

Supplementary Figure 2. Bone marrow–derived APCs display stimulus-dependent phagocytic and pinocytic activity. (a) Representative flow-cytometric gating strategies for phagocytosis in bmMΦs and bmDCs under three conditions each: Mock, LPS stimulation, and 4°C negative control (total of six panels). **(b)** Equivalent gating strategies for pinocytosis under the same conditions. **(c)** Representative confocal microscopy images of bmAPCs incubated with IgG–FITC beads at 4°C as a non-internalizing control. **(d)** Representative confocal images of bmAPCs incubated with FITC–Dextran at 4°C.

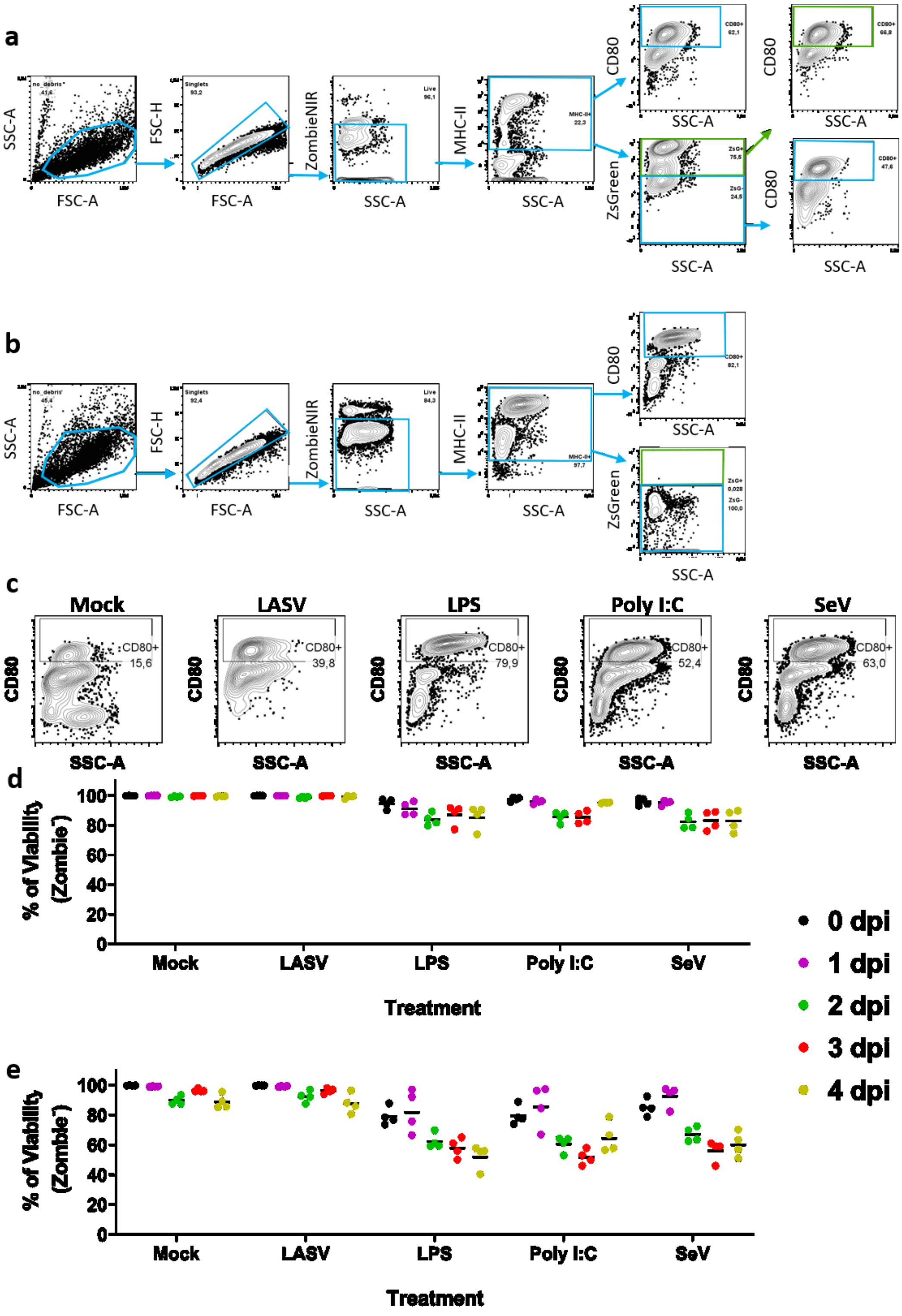

Supplementary Figure 3. LASV infection does not alter cell viability but selectively induces CD80 upregulation in dendritic cells. (a) Representative gating layout for LASV-infected samples, illustrating the additional ZsGreen-based branch incorporated into the analysis workflow. **(b)** Representative gating layout for non-infected controls, showing the corresponding strategy in the absence of a ZsGreen gate. **(c)** Contour plots depicting the final gating step used to define mature APCs (Zombie^{-}/MHC-II^{+}/CD80^{high}), with one representative CD80 vs. SSC-A plot shown for each treatment condition. **(d)** Flow-cytometric analysis of viability (Zombie^{-} cells) in bmMΦs over a 4-day time course following LASV-ZsGreen infection (MOI = 2) or stimulation with LPS, Poly I:C, or SeV. **(e)** Equivalent viability analysis in bmDCs. Data represent four animals, and lines indicate arithmetic means.

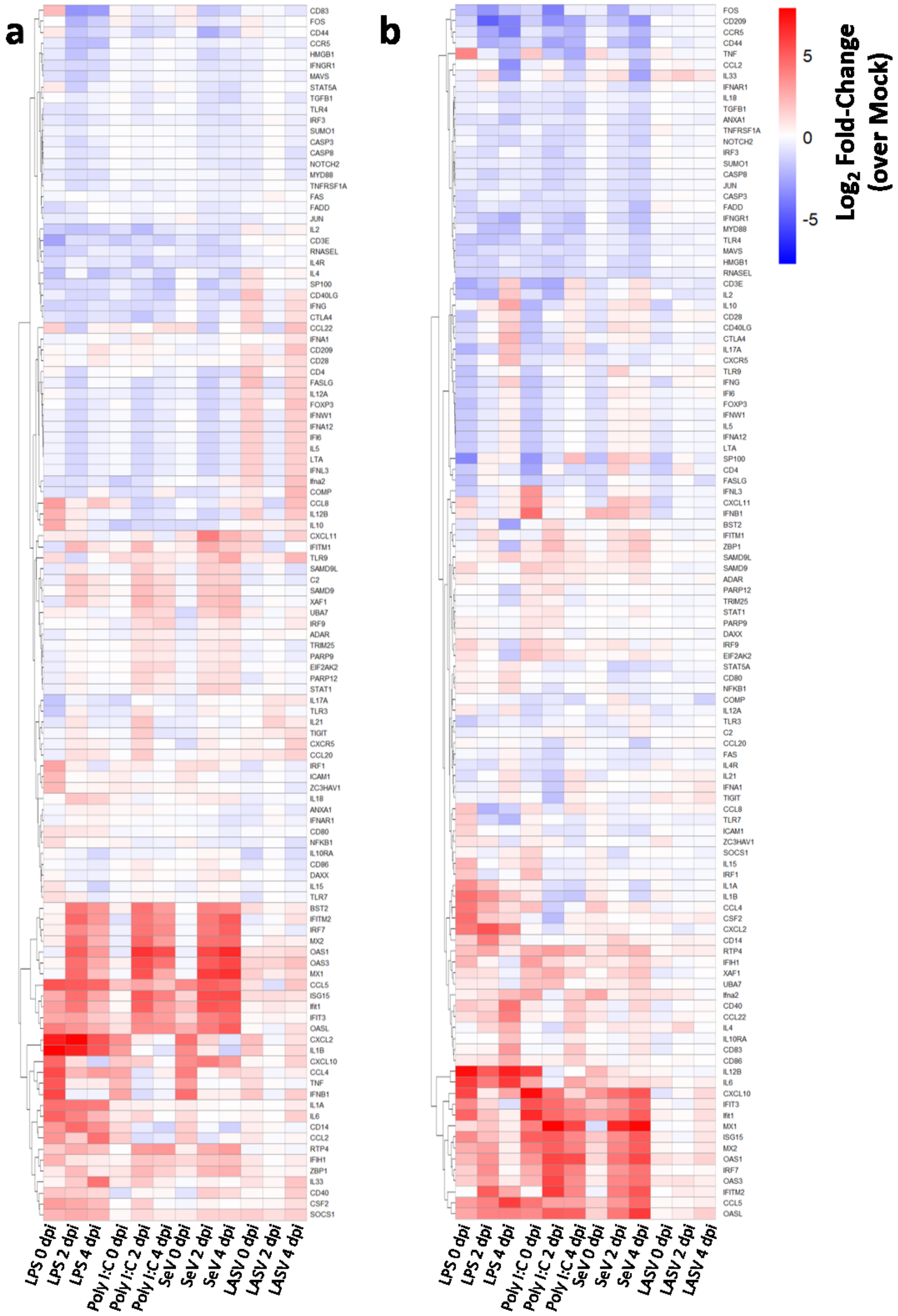

Supplementary Figure 4. Extended differential expression heatmaps for bmMΦs and bmDCs across all treatments and time points. (a) Heatmap of all $\log_2$ fold-changes for the 143-gene

NanoString panel in bmMΦs following LASV infection or stimulation (LPS, Poly I:C, SeV) at 2 and 4 DPI, calculated using the limma-voom pipeline. All detected genes are shown without significance masking, providing a complete overview of transcriptional amplitude in bmMΦs. **(b)** Equivalent all $\log_2$ fold-change heatmap for bmDCs. Together, panels A and B highlight the overall transcriptional architecture of each cell type, independent of statistical thresholds. All heatmaps display mock-normalized $\log_2$ fold-changes; colors indicate the direction and magnitude of differential expression, and genes appear in the same order across all panels
